# Annotations

**Published:** 1889-10-12

**Authors:** 


### Annotations.

**Smokeless London.** The season of mist and mellow fruitfulness means, for the dwellers in towns, the season of fog. Sulphurous particles, issuing from chimneys, mix themselves with the morning vapours and form a compound of which we shall have more than enough a month hence. Whoever could deliver us from the fog-demon would deeply benefit us poor mortals, not to speak of the longer life and heightened beauty that would be accorded our statues and public buildings if they could be freed from their coating of soot. If the experiments recently held at Willis's Rooms may be trusted, the thing can be done, and without any of the expensive and elaborate apparatus which frightens the British soul, and makes us hesitate at evading the ills we have, lest we fly to others in the way of danger and expense that as yet we know not of. The cost of rendering a ton of coal smokeless by the new process is sixpence, and this is more than compensated for by the fact that the coal thus treated burns as long as 25 cwt. left in its natural condition. A saving of 25 per cent. on the coal-bill is not to be despised now that winter is coming on. The process is simplicity itself. A solution is made of a grey crystalline powder, and into this the coal is dipped, and allowed to remain till the powder settles in a thin grey crust upon its surface. It is then fit to burn, which it does, with a clear flame and a continual glow. When the coal is first put on there arises a slight grey vapour, which, however, is not smoke, as may be tested by anyone who will put his hand in the midst of it. He will withdraw it unsoiled, and will perceive only a pungent odour of burning wood hovering about it. The inventors claim that their solution fixes all the constituents of coal in such a fashion that all burn, and none are mischievously wasted in smoke. Thence arises both the comfort and the economy we speak of. Certain it is that a fire made of their coal burns with great clearness and heat, with no smoke and very little ash. The coal merchants are said to look favourably on the scheme, and if they adopt it we hope yet to have a smokeless London, pure and healthy.

**Half Madness and Responsibility.** We publish elsewhere a brief communication on this subject from the Rev. P. H. Good, M.A., chaplain to the Borough of Portsmouth Lunatic Asylum. From a psychological point of view the question is profoundly interesting, and eminently worthy of scientific consideration. It is rather, however, to its practical aspect that we wish to draw attention at the present time. It need not be doubted for a moment that, with certain persons, there is a condition between sleeping and waking when the mental faculties are by no means in a state of normal and rational activity. The probability, indeed, is that practically all people—men, women, and children—are for a longer or shorter time, when newly aroused from sleep, in a state of defective mentality. With some the conditions may not last more than a few seconds, with others it may continue for several minutes. Children offer excellent practical examples of what is meant. When they are roused in the morning, they will sometimes rub their eyes slowly, look about them with a dazed expression, and seem as if they could not decide whether to wake up fully or to go to sleep again. At other times they will open their eyes and appear quite bright and lively all at once. Another frequent condition with children on waking is one of crossness and irritability. They cannot control their tempers for a long time, so that the nurse will say they have "got out on the wrong side of the bed." Nurses and mothers, and especially stern maiden aunts, are always ready to denounce such morning tempers as "wicked," and to prescribe a dose of "whipping" as a remedy. It is you who would be "wicked," if it were not that you are merely foolish and ignorant maiden aunts! The child who wakes up "cross" has had his brain disturbed and irritated in his sleep. If you ask—what by? it is impossible to say positively. Perhaps by a dream, perhaps by the chocolates or the jam-tart he ate before going to bed. But whatever the cause, the fact is that the child's brain was irritated before he awoke cross, and he is not to be blamed but pitied, and helped out of his crossness by rational means. A little hot milk and water to drink may be what he requires, or if the weather be hot a little fresh cold water. Perhaps all he needs is time for the cerebral blood to resume its full activity. A brief, bright conversation or a funny toy may help him. But to be angry with him or to whip him is ignorant, stupid, and unjust, and can only make matters worse in every way. That is an example which all the world is familiar with. Exactly the same kind of thing may happen to grown-up people as the result of irritation of the brain in sleep; and it may just as easily be induced by a bad dream or a heavy supper, as it was in the case of the child. The real importance of the subject is, however, in its relation to epileptics, half-lunatics, and unfortunate creatures of that kind. Many a person who suffers from slight epileptic seizures —*le petit mal*, of the French—is held by the uninstructed to be quite as accountable as the sanest of sane men. Yet there are, undoubtedly, periods in the history of all such epileptics when they might commit the most monstrous crimes without being responsible at all. It is exactly so with many persons who are on the border-land of madness. They may be sane for twenty-three hours of the day, and yet quite irresponsible in the twenty-fourth. They may be as rational as possible for weeks together, and quite insane and irresponsible for days thereafter. The writer has more than once known a man perform all the duties of a sane and responsible citizen for months together, who was alternately mad and sane in this way, and who finally lapsed into complete imbecility. What, then, of the all-important question of responsibility? There is only one way of dealing with such persons. Their past lives must be taken into full and careful review, and the question must be decided on these lines. A man who has been wise and sober until fifty years of age is not likely to become frivolous and inconsequent after that time, unless his brain break down. A woman who has been virtuous through youth and mid-age will not become vicious in old age, except as the result of structural changes in her cerebrum. The whole question is deeply and perennially important, and the formation of a sound public opinion upon it is an urgent practical necessity.

**The Barrister's Tea.** The reign of politics is temporarily suspended and all sorts of social questions, small and great, are lifting up their heads for a season. Among the rest the great "tea question" is having its share of public regard. Sir Andrew Clark, a considerable authority, puts his trust in "five minutes' tea," or tea infused exactly five minutes, with boiled water. But the medical profession has for once been beaten on its own ground, and a barrister carries off the honours of the day. The gentleman in question, following up a succession of correspondents in a daily contemporary, describes a method of making tea which we know to be practically one of the best possible. It secures the full strength and pure flavour of the leaf without extracting more than the smallest minimum of the undesirable tannin. This is the barrister's easy, artless, and inexpensive way: Put the kettle on to boil; when the water absolutely boils fill *two* teapots, containing no tea, with it in the boiling condition; let the two pots stand until they are both thoroughly hot; then empty one of them, and put for every pint of water the pot will hold a single teaspoonful of good tea. The barrister uses an Indian mixture at 3s. 6d. per lb. Place the teapot, filled with water and the requisite number of spoonsful of tea, on a stove or hob for exactly five minutes. It is then sufficiently infused. The other teapot which has been heated by the boiling water is now emptied, and the infused tea is all poured out of pot No. 1 into it, care being taken to keep the leaves entirely separate. Pot No. 2, which contains the infused tea, minus the leaves, is now fit for use; and in addition to being a perfect brew, perfectly hot, it has this advantage that it can be kept hot, and allowed to stand for one hour, two hours, or even six, without spoiling or becoming any stronger, for the simple reason that all the leaves were left behind in the first pot. If a "Barrister's" methods of law be as rational as his method of making tea, he probably has a brilliant career before him.

